# Prognostic Implications of CD10 and CD15 Expression in Papillary Thyroid Carcinoma

**DOI:** 10.3390/cancers12061413

**Published:** 2020-05-30

**Authors:** Eun Ji Oh, Andrey Bychkov, Haejin Cho, Tae-Min Kim, Ja Seong Bae, Dong-Jun Lim, Chan Kwon Jung

**Affiliations:** 1 Department of Hospital Pathology, College of Medicine, The Catholic University of Korea, Seoul 06591, Korea; ejo0805@naver.com; 2 Cancer Research Institute, College of Medicine, The Catholic University of Korea, Seoul 06591, Korea; haejincho59@catholic.ac.kr (H.C.); tmkim@catholic.ac.kr (T.-M.K.); drbae@catholic.ac.kr (J.S.B.); ldj6026@catholic.ac.kr (D.-J.L.); 3 Department of Pathology, Green Cross Laboratories, Yongin-si, Gyeonggi-do 16924, Korea; 4 Department of Pathology, Kameda Medical Center, Kamogawa, Chiba 296-8602, Japan; bychkov.andrey@kameda.jp; 5 Department of Pathology, Nagasaki University Graduate School of Biomedical Sciences, Nagasaki 852-8523, Japan; 6 Department of Medical Informatics, College of Medicine, The Catholic University of Korea, Seoul 06591, Korea; 7 Department of Biomedicine & Health Sciences, Graduate School, The Catholic University of Korea, Seoul 06591, Korea; 8 Department of Surgery, College of Medicine, The Catholic University of Korea, Seoul 06591, Korea; 9 Department of Endocrinology and Metabolism, Department of Internal Medicine, College of Medicine, The Catholic University of Korea, Seoul 06591, Korea

**Keywords:** papillary thyroid carcinoma, CD10, CD15, recurrence, radioactive iodine, prognosis, biomarker, therapeutic response

## Abstract

Patients with papillary thyroid carcinoma (PTC) have excellent survival, but recurrence remains a major problem in the management of PTC. We aimed to determine the prognostic impact of the expression of CD10 and CD15 in patients with PTC. Immunohistochemistry for CD10 and CD15 was performed on the tissue microarrays of 515 patients with PTC. The expression of CD10 and CD15 was detected in 201 (39.0%) and 295 (57.3%) of 515 PTC cases, respectively, but not in the adjacent benign thyroid tissue. Recurrence was inversely correlated with CD15 expression (*p* = 0.034) but not with CD10 expression. In 467 PTC patients treated with radioiodine remnant ablation, the CD15 expression had an adjusted hazard ratio of 0.500 (*p* = 0.024) for recurrence-free survival and an adjusted odds ratio of 2.678 (*p* = 0.015) for predicting long-term excellent therapeutic response. CD10 expression was not associated with clinical outcomes. In the Cancer Genome Atlas dataset, the expression level of *FUT4* (*CD15*) mRNA was higher in the low/intermediate-risk group for recurrence than in the high-risk group and exhibited positive correlation with *SLC5A5* (*NIS*) mRNA expression (*p* = 0.003). Taken together, CD15 expression was identified as an independent prognostic marker for improved prognosis in PTC patients.

## 1. Introduction

Papillary thyroid carcinoma (PTC) is the most common histologic type of thyroid cancer, accounting for 86% in the USA and 93% in Korea and Japan [1,2,3]. Most of the patients with PTC are not likely to die of this disease. The 5-year survival rate has been reported as 97.9% in the USA and 100% in Korea [1,4]. However, up to 20% of patients with PTC have a locoregional recurrence or distant metastasis following thyroidectomy [5]. The 2015 American Thyroid Association (ATA) guidelines classify patients with differentiated thyroid cancer into low, intermediate, and high-risk groups for recurrence after initial complete therapy based on the specific histology, tumor size, tumor encapsulation, multifocality, the extent of extrathyroidal extension, number of vascular invasion foci, number and size of the metastatic lymph nodes, and mutational status of *BRAF^V600E^* and/or *TERT* promoter when available [6]. However, the ATA three-tiered system is unable to accurately predict the likelihood of recurrence in an individual patient. Increasing knowledge of tumor biomarkers has led to several efforts to more accurately predict the recurrence risk for thyroid cancer patients.

The cluster of differentiation (CD) antigens has gathered increased recognition not only as diagnostic biomarkers but also as a target for molecular therapy. A wide range of CD markers was initially described in various immune cells and hematological malignancies [7]. Later on, a spectrum of tissues expressing CD antigens was expanded to various solid tumors. We recently described the aberrant expression of CD20 in thyroid cancer, particularly the aggressive types, which may offer promise for translational implications [8]. This finding prompted us to further explore CD markers in thyroid tumors.

CD10, also known as membrane metalloendopeptidase (MME), neprilysin, common acute lymphoblastic leukemia antigen (CALLA), neutral endopeptidase (NEP), enkephalinase, or EC 3.4.24.11, is a membrane-bound zinc metalloproteinase [9,10]. Immunohistochemistry for CD10 has been used for diagnostic purposes in lymphomas, leukemias, and solid tumors including clear cell renal cell carcinoma, solid and pseudopapillary neoplasm of the pancreas, skin tumors, urothelial tumors, endometrial stromal tumors, and mesonephric tumors [10]. Conflicting results have been reported for the association of altered expression of CD10 with adverse prognosis in various solid tumors including lung cancer, prostate cancer, head and neck cancer, colorectal cancer, melanoma, and ovarian cancer [9,10]. Several previous studies also reported selective expression of CD10 in differentiated thyroid cancers and not in normal thyroid tissues and benign thyroid nodules [11,12,13]. It has recently been reported that CD10 is highly expressed in anaplastic thyroid carcinoma, but has low expression in PTC and is absent in follicular thyroid carcinoma and medullary thyroid carcinoma [14]. However, these studies were performed on a relatively small number of cases. Little is known about the diagnostic and prognostic values of CD10 expression in PTC.

CD15, also known as fucosyltransferase 4 (FUT4), LeuM1, Lewis X, or stage-specific embryonic antigen 1 (SSEA-1), is a carbohydrate antigen with the common trisaccharide structure 3-fucosyl-N-acetyl-lactosamine [15]. CD15 is a marker for human granulocytes and Reed–Sternberg cells of Hodgkin’s lymphoma [16]. CD15 expression has also been found in various solid cancers including lung cancer [17], breast cancer [18], colorectal cancer [19,20], ovarian cancer [21], and renal cell carcinoma [22,23]. In the thyroid gland, CD15 expression was reported in various types of thyroid cancers, but not in normal thyroid tissue or benign thyroid neoplasms [24]. CD15 was suggested as a marker for thyroid cancer-initiating cells or cancer stem cells [24,25,26].

This study aims to investigate the prognostic potential of the expression of CD10 and CD15 by immunohistochemistry in a large series of PTC. Furthermore, the correlation between mRNA expression of both the markers and clinicopathologic features using The Cancer Genome Atlas (TCGA) data was examined, and in silico gene expression analyses of the related genes were performed.

## 2. Results

### 2.1. Baseline Characteristics

The clinicopathologic characteristics of patients and the association between clinicopathologic parameters and CD10 and CD15 expression are summarized in Table 1. A total of 515 PTC cases were studied consisting of 385 classic PTCs, 68 classic PTCs with tall cell features, 17 follicular variant PTCs (including five invasive encapsulated follicular variants and 12 infiltrative follicular variants), 21 tall cell variant PTCs, and 24 PTCs of other variants (11 oncocytic, eight Warthin-like, two hobnail, one solid, one diffuse sclerosing, and one cribriform-morular).

### 2.2. Clinicopathologic Significance of CD10 and CD15 Protein Expression

**Figure 1:**
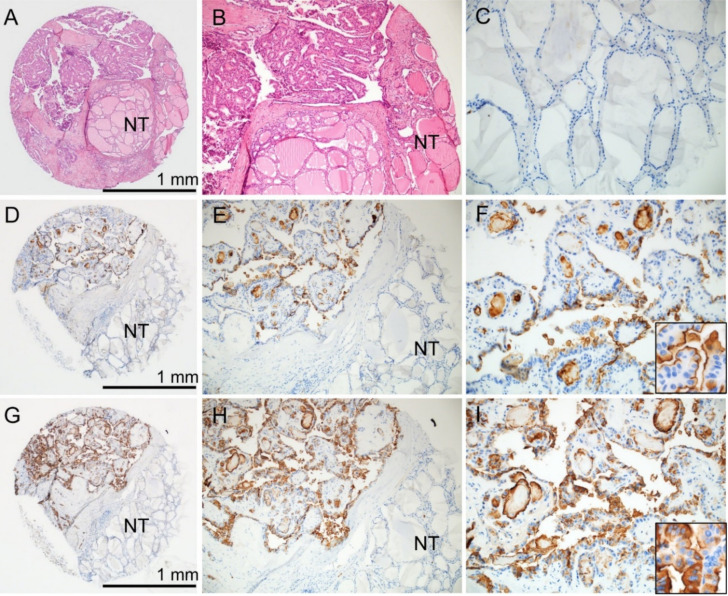
Immunohistochemical staining for CD10 and CD15 on the tissue microarray of papillary thyroid carcinoma. (**A**) The tissue core contains tumor and non-tumor (NT) areas (hematoxylin and eosin stain). (**B**) The higher power view of the tissue core shows papillary thyroid carcinoma and NT adjacent to tumor. (**C**) NT in the same case is negative for both CD10 and CD15. Tumor cells in the same case are positive for both CD10 (**D**–**F**) and CD15 (**G**–**I**) immunostaining. (**F**) Inset shows CD10 staining at the apical surface of tumor cells. (I) Inset shows CD15 cytoplasmic and membranous staining in tumor cells; ×40 (**A**,**D**,**G**), ×100 (**B**,**E**,**H**), ×200 (**C**,**F**,**I**), inset (×400).

Immunohistochemical staining for CD10 showed membranous staining at the apical surface of tumor cells (Figure 1). The staining pattern for CD15 was both cytoplasmic and membranous in tumor cells (Figure 1). Normal thyroid tissue adjacent to tumors was negative for both CD10 and CD15 staining. Positive staining of CD10 and CD15 was found in 201 (39.0%) and 295 (57.3%) of 515 PTC cases, respectively.

The relationship between clinicopathological characteristics and the expression of CD10 and CD15 is shown in Table 1. The expression rates of CD10 and CD15 were higher in female patients (*p* = 0.049 and *p* = 0.009, respectively). Tall cell variant PTC had higher positive rates of CD10 and CD15 than classic PTC and follicular variant PTC. Compared with patients without extrathyroidal extension, patients with minimal (microscopic) extrathyroidal extension had higher positive rates of CD10 and CD15 (*p* = 0.003 and *p* = 0.014, respectively). However, the expression of CD10 and CD15 was not associated with gross extrathyroidal extension, pathologic T stage, or lymph node metastasis. *BRAF*^V600E^ mutation was significantly associated with the expression of CD10 and CD15 (*p* = 0.011 and *p* < 0.001, respectively). The rate of structural recurrence was lower in patients with CD15 expression than those without CD15 expression (*p* = 0.034) but was not associated with CD10 expression (*p* = 0.656). Distant metastases developed in 11 patients as synchronous (n = 3) or metachronous (n = 8) lesions. Although not statistically significantly different, CD15 expression was less frequently found in patients with distant metastasis (*p* = 0.062).

### 2.3. Analysis of Recurrence-Free Survival

For the analysis of recurrence-free survival, nine patients with initial distant metastasis (*n* = 3) or less than six months of follow-up data (*n* = 6) were excluded from the study. The median follow-up time was 112 months (range 6–143 months). During the follow-up, structural recurrence occurred in 44 (8.7%) of 506 patients. The median time to the first recurrence was 15 months (range, 6–23 months). The recurrence occurred in 43 (9.2%) of 467 patients who underwent surgery and radioactive iodine (RAI) remnant ablation whereas the incidence was noted in one of 48 patients who were not indicated for the RAI therapy. Therefore, the analysis of recurrence-free survival was performed in 467 patients who underwent total thyroidectomy and RAI remnant ablation.

**Figure 2:**
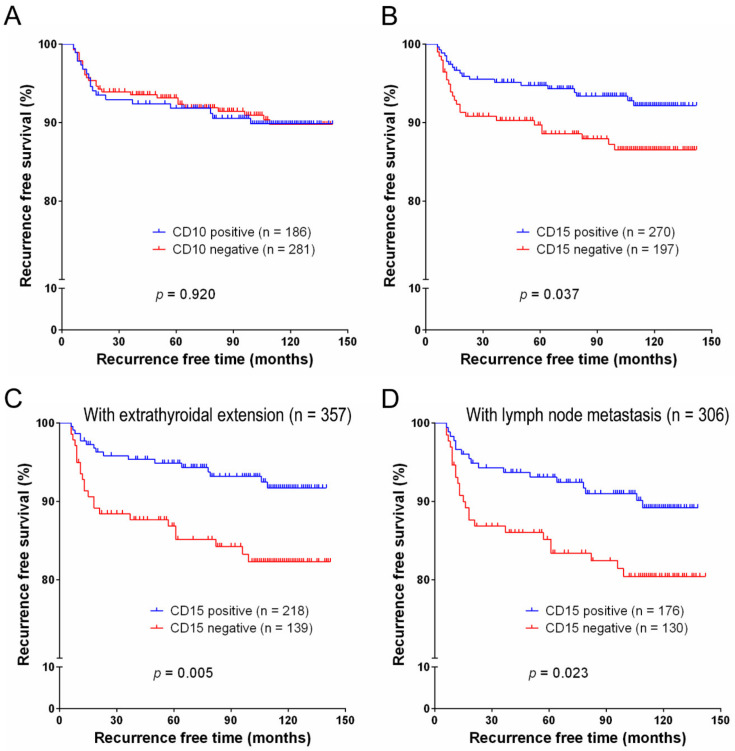
Recurrence-free survival analysis of CD10 and CD15 expression in 467 patients with papillary thyroid carcinoma treated with total thyroidectomy and radioiodine remnant ablation. The recurrence-free survival was not correlated with CD10 expression (**A**) but was significantly correlated with CD15 expression (**B**). Stratified survival analyses show a significant association between CD15 expression and recurrence-free survival in patients with minimal and gross extrathyroidal extension (**C**) and lymph node metastasis (**D**).

On univariate survival analysis, pT3-4 stage (*p* = 0.022) and lymph node metastasis (*p* < 0.001) were significantly associated with decreased recurrence-free survival. The expression of CD15 was associated with increased recurrence-free survival (*p* = 0.037) (Figure 2). In the stratified survival analyses, the expression of CD15 was also a predictor for the increased recurrence-free survival in subgroups of PTC patients with extrathyroidal extension (*n* = 357, *p* = 0.005) and lymph node metastasis (*n* = 306, *p* = 0.023) (Figure 2). However, CD10 expression was not correlated with recurrence-free survival (*p* = 0.920).

Multivariate analysis revealed an independent correlation between lymph node metastasis and poor recurrence-free survival (*p* = 0.001). Patients with CD15 expression had an adjusted hazard ratio of 0.500 (95% confidence interval (CI): 0.274–0.911, *p* = 0.024) (Table 2).

### 2.4. Therapeutic Response to RAI

The clinical outcomes in 467 patients who underwent total thyroidectomy and RAI remnant ablation were further analyzed. At the time of the last follow-up, 438 (93.8%) patients achieved an excellent response and were considered to have no clinical evidence of disease (NED). All patients without lymph node metastasis achieved an excellent response.

On univariate logistic regression analysis, NED was significantly associated with lower pT stage (*p* = 0.006) and the presence of CD15 expression (*p* = 0.029) (Table 3). On multivariate logistic regression analysis, patients with CD15 expression had an adjusted odds ratio of 2.678 (95% CI: 1.215–5.902, *p* = 0.015) for predicting NED compared to those without CD15 expression (Table 3).

### 2.5. Clinicopathologic Significance of MME and FUT4 mRNA Expression in TCGA Dataset

A high level of *MME* (*CD10*) mRNA expression was associated with minimal extrathyroidal extension (*p* < 0.001), pathologic T stage (*p* = 0.006), lymph node metastasis (*p* < 0.001), and advanced cancer stage (*p* = 0.007). A high level of *FUT4* (*CD15*) mRNA expression was associated with younger age (<45) (*p* = 0.001) and histologic variant (*p* = 0.031), as shown in Table 4. PTCs with a *BRAF*-like molecular phenotype had higher levels of *MME* and *FUT* mRNA than did *RAS*-like tumors (*p* < 0.001 and *p* = 0.002, respectively). According to the ATA recurrence risk stratification, the expression level of *MME* mRNA expression was higher in the intermediate/high-risk group than in the low-risk group (*p* < 0.001) whereas the expression level of *FUT4* mRNA expression was higher in the low/intermediate-risk group than in the high-risk group (*p* = 0.023).

### 2.6. Relationship between FUT4 (CD15) mRNA Expression and the Tumor Microenvironment

**Figure 3:**
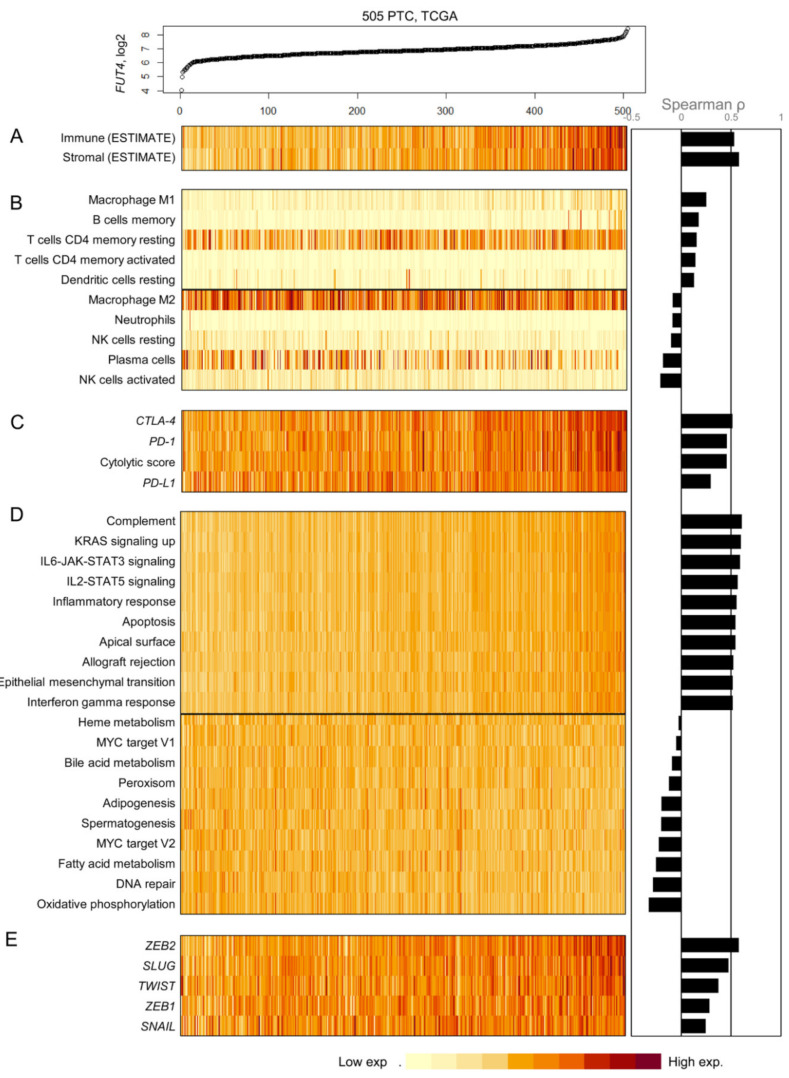
Correlation between *FUT4* (*CD15*) expression levels and expression patterns of tumor microenvironment-related genes in 505 papillary thyroid carcinomas (PTCs) from the TCGA cohort. (**A**) The expression of *FUT4* (*CD15*) mRNA is positively correlated with the infiltration level of immune and stromal cells based on the estimation by the ESTIMATE algorithm. The relative abundance of tumor infiltrating immune cells analyzed by the CIBERSORT algorithm (five cell types with positive correlation and five cell types with inverse correlation with *FUT4* expression selected) (**B**), the expression level of immune checkpoints and cytolytic score (**C**), single sample enrichment score of MsigDB hallmark gene sets (10 positive and 10 inverse correlations with the selected *FUT4* expression) (**D**), and epithelial mesenchymal transformation-associated genes (**E**).

Since CD15 showed clinical and prognostic significance in PTC based on protein and mRNA expression in the two independent datasets, as described above, we further addressed the possible relationship between the *FUT4* (*CD15*) mRNA expression and tumor microenvironment in PTC. The gene expression profiles of 505 primary PTCs were obtained from the TCGA project (Figure 3). Initially, the level of infiltration for immune and stromal cells was measured by ESTIMATE (Estimation of STromal and Immune cells in MAlignant Tumor tissues using Expression data) algorithm [29]. A substantial level of correlation with *FUT4* mRNA expression was observed, i.e., Spearman correlation of 0.532 and 0.578 for immune and stromal cells, respectively (Figure 3A). These findings suggest that *FUT4* expressing PTCs are highly infiltrated with immune and stromal cells.

To further examine the immune cell composition concerning *FUT4* mRNA expression, the CIBERSORT algorithm was employed [30]. The relative abundance of CD4 memory T cells, B cells, and dendritic cells was positively correlated with *FUT4* mRNA expression while that of neutrophils, plasma cells, and NK (Natural Killer) cells showed inverse correlation (Figure 3B). Of note, distinct macrophage polarization with respect to *FUT4* mRNA expression was observed; macrophages M1 and M2 showed a positive and inverse correlation with *FUT4* mRNA expression, respectively, suggesting that high *FUT4* mRNA expression favors the infiltration of antitumorigenic M1 macrophages in PTC microenvironments.

The mRNA expression levels of three immune checkpoint markers (*PD-1*, *PD-L1*, and *CTLA-4*) were further examined with the cytolytic score as the geometric mean of *GZMA* and *PRF1* mRNA expression (Figure 3C); all the parameters demonstrated a substantial level of correlation with *FUT4* mRNA expression. This is suggestive of the high level of infiltration of cytolytic T lymphocytes (cytolytic score) and their exhaustive states with a high level of expression of immune checkpoints. Gene set enrichment analysis of 50 hallmark gene sets revealed a positive correlation between immune-related gene set scores and *FUT4* mRNA expression, consistent with ESTIMATE results. In addition, a positive correlation between inflammatory response and expression of epithelial-mesenchymal transition (EMT) associated genes, explaining the high level of infiltration of stromal cells with high *FUT4* mRNA expression in PTC, was observed (Figure 3D). Consistently, transcription factors known to promote EMT such as *TWIST* and *SLUG* showed a high level of correlation with *FUT4* mRNA expression (Figure 3E). These findings suggest that *FUT4* mRNA expression in PTC might be associated with distinct immune and stromal composition of the tumor microenvironment in PTC.

### 2.7. Relationship between FUT4 (CD15) and SLC5A5 (NIS) mRNA Expression

**Figure 4:**
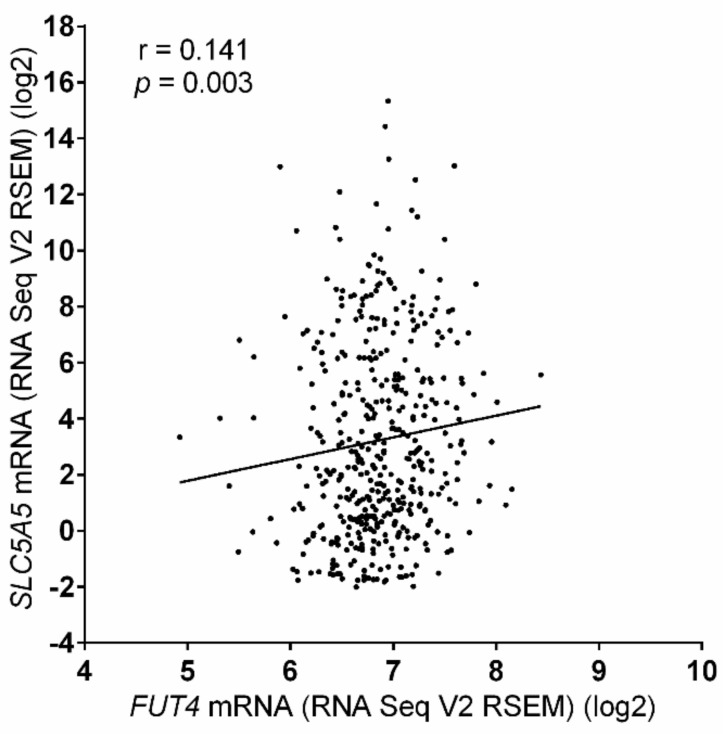
Correlation between mRNA expression of *FUT4* and *SLC5A5* in the TCGA database.

From the above results, initially it was hypothesized that CD15 overexpression might be associated with poor prognosis in PTC patients because it was associated with some unfavorable clinicopathological variables (aggressive histology, minimal extrathyroidal extension, and *BRAF*^V600E^) in our study cohort. However, patients with CD15 expression were more responsive to RAI therapy and had better recurrence-free survival compared to those without CD15 expression. To address the contradictory results and explain the high therapeutic response rate in patients with CD15 expression, the correlation between expression levels of *FUT4* and *SLC5A5* mRNA in the TCGA cohort was analyzed. Recently, it was demonstrated that the *SLC5A5* mRNA expression is more reliable than immunohistochemical expression of sodium iodide symporter (NIS) coded by *SLC5A5* gene regarding tumor behavior, therapeutic response, and prognostic outcomes [31]. Herein, a positive correlation between the two variables (Spearman Rho = 0.141, *p* = 0.003) was observed, as shown in Figure 4.

## 3. Discussion

In this study, the prognostic implication of CD10 and CD15 expression was investigated in PTC patients. The patients with CD15 expression had a good recurrence-free survival at about the 10 years median follow-up and excellent therapeutic outcomes after total thyroidectomy and RAI remnant ablation for PTC. However, CD10 expression was not identified to be associated with clinical outcomes.

Overexpression of the *FUT4* gene has been reported in different malignancies; however, the exact role of *FUT4* expression in carcinogenesis still remains unclear. Many previous studies reported that *FUT4* gene expression is associated with pro-tumorigenic function, such as tumor growth and invasion, and drug resistance [32]. In contrast, *FUT4* overexpression in lung cancer cells has been reported to suppress the EGFR signaling pathway and attenuate EGFR-mediated invasion of tumor cells [33].

In our study, we found that high expression of *FUT4* mRNA in PTC from the TCGA database was associated with a high level of tumor infiltration both for immune and stromal cells as exhibited by ESTIMATE analysis. *FUT4*-overexpressing PTCs showed transcriptional up-regulation of EMT-associated genes (*ZEB2*, *SLUG*, *TWIST*, *ZEB1*, and *SNAIL*). Therefore, *FUT4*-overexpressing PTCs may have activated EMT during their progression and are also highly infiltrated with stromal cells. However, recent studies suggest that transcriptional signals of mesenchymal tumor subtypes largely come from stromal cells instead of tumor cells, and EMT-like tumor signatures may be largely determined by tumor purity [27,34]. Therefore, it will require further investigation to see whether the *FUT4*-overexpressing PTCs activate EMT or represent those with a high level of tumor-infiltrating stromal cells and low tumor purity.

In terms of immune cells, we observed that *FUT4*-overexpressing PTCs had a high level of M1 macrophages infiltration and depleted M2 macrophages. The polarization of M1 and M2 macrophages with immunostimulatory and immunomodulatory phenotypes, respectively, define key components of tumor microenvironments [35]. Therefore, the correlation of *FUT4* expression with M1 and M2 macrophages is suggestive of the immune microenvironment exhibiting antitumor activities associated with *FUT4* expression. The cytolytic scores estimated from the transcript level of two key cytolytic effectors of *GZMA* and *PRF1* [36] also showed the correlation with *FUT4* expression, suggesting that the *FUT4*-overexpressing PTCs are likely to be infiltrated with cytotoxic T cells. In contrast, *FUT4*-overexpressing PTCs were associated with up-regulation of *CTLA-4*, *PD-1*, and *PD-L1* in the tumor microenvironment that is known to exert immunosuppressive and pro-tumorigenic activities [37]. CTLA-4 and PD-1 negatively regulate T-cell activation and alter the motility and migration of T-cells [37]. Therefore, *FUT4*-overexpressing PTCs could possess the immune signatures indicating T cell exhaustion (i.e., high expression level of immune checkpoints) as well as activated T cell effectors (i.e., high level of cytolytic scores).

However, despite the potential cancer-promoting effects in PTC, the CD15 expression was found to be an independent prognostic predictor for reduced disease recurrence and excellent therapeutic response in our original institutional cohort. As most of the PTC patients at our hospital underwent total thyroidectomy and RAI remnant ablation in accordance with a standard protocol during the study period, we could not analyze the long-term prognosis and therapeutic response rate according to the CD15 expression status in a subgroup of PTC patients without RAI therapy. To address the question about excellent therapeutic response in patients with CD15 expression, the gene expression patterns of CD15 and NIS were compared using the TCGA data, and positive correlation was observed between expression levels of both the genes. Therefore, it is hypothesized that good response to RAI therapy in PTC patients with CD15 expression might be associated with increased expression of *SLC5A5* mRNA.

The prognostic value of positive expression of CD15 for the clinical outcome in thyroid cancer patients remains controversial. In one previous study based on tissue microarray, CD15 immunostaining was usually focal in PTC and follicular thyroid carcinoma but diffuse in anaplastic thyroid carcinoma [24]. CD15 expression was associated with worse survival in anaplastic thyroid carcinoma, but not in PTC or follicular thyroid carcinoma [24]. In another report, CD15 expression was associated with recurrence or metastasis and shorter progression-free survival [25]. However, limitations of both these studies included a relatively small number of cases, selection bias, short-term follow-up, and different treatment strategies and follow-up methods. In our study, CD15 expression was associated with minimal extrathyroidal extension and *BRAF*^V600E^ mutation, but not with gross extrathyroidal extension and lymph node metastasis, which are more important parameters for prognosis in PTC [38].

CD15 expression was independently associated with improved recurrence-free survival and excellent therapeutic response when we analyzed PTC patients who underwent total thyroidectomy with RAI remnant ablation and thyroid-stimulating hormone (TSH) suppression. In the TCGA dataset, the expression levels of *FUT4* (*CD15*) mRNA were higher in the low (47.0%) and intermediate (53.2%) recurrence risk groups compared to that in the high-risk group (25%). These results therefore indicate that high expression of CD15 protein and mRNA may represent prognostic markers that predict favorable clinical outcomes of patients after the treatment of PTC. A better understanding of the implications of CD15 expression may be crucial for developing biomarkers for monitoring and treating PTC patients.

Previous studies have reported different prognostic values of CD15 expression in various solid tumors besides thyroid cancers. In Chinese patients with clear cell renal cell carcinoma, CD15 expression was associated with improved overall survival after surgical treatment [23]. In Japanese patients who underwent radical nephrectomy for renal cell carcinoma, high CD15 expression was associated with recurrence and shorter metastasis-free survival [22]. In metastatic colorectal cancer patients treated with cetuximab or bevacizumab plus chemotherapy, CD15 expression in cancer cells was associated with worse progression-free survival and overall survival [19]. Therefore, it is conjectured that CD15 may play various roles in solid cancer development and progression depending on the type of cancer.

Until now, only limited data were available on the role of CD10 in thyroid carcinoma. Most of the previous studies focused on the diagnostic significance of CD10, for example, they were aimed at differentiatimg thyroid cancers from benign thyroid lesions [11,12,13,14]. The positivity of CD10 has been reported to be significantly higher in PTC than in benign thyroid tumors, but lower than in anaplastic thyroid carcinoma [11,12,14]. Anaplastic thyroid carcinoma shows diffuse positivity for CD10, whereas most of the cases of PTC had focal expression of CD10 [13,14]. Anaplastic thyroid carcinoma can develop from differentiated thyroid carcinoma through the stepwise process of dedifferentiation and can involve accumulation of genetic abnormalities [39]. PTC is the most common type of differentiated thyroid carcinoma in the case of anaplastic thyroid carcinomas comprising both the tumor components [40]. In the present study, we observed the focal staining pattern for CD10 in PTC cells. The expression of CD10 was significantly associated with minimal extrathyroidal extension (but not with gross extrathyroidal extension) and *BRAF*^V600E^ mutation. TCGA data showed similar results with high expression of *MME* (*CD10*) mRNA being associated with minimal extrathyroidal extension and a pathologic T stage as classified by the American Joint Committee (AJCC) 7th edition and *BRAF*-like molecular signature. However, analysis of long-term follow-up in our study cohort showed that the expression of CD10 was not associated with tumor recurrence and clinical outcome of PTC patients. *BRAF*^V600E^ mutation is an early event in the development of PTC [28,41,42]. Apparently, these findings indicated that CD10 overexpression might be a relatively early event in the development and progression of PTC. As CD10 expression has been reported to be associated with cancer progression and chemotherapy susceptibility in other cancers [9], our findings still merit further experimental investigations.

## 4. Materials and Methods

### 4.1. Patients

A total of 515 patients who underwent surgery for PTC with tumor size ≥1 cm at Seoul St. Mary’s Hospital from 2008 to 2010 were enrolled in this study. The institutional review board approved this study (KC16SISI0104 and KC16SISI0709) and all the patients provided informed consent. Clinical information was obtained from the medical records. All hematoxylin and eosin slides of thyroidectomy specimens were reviewed by an endocrine pathologist (Chan Kwon Jung). In cases of multiple tumor foci, the largest one was considered as the index lesion. All cases were classified according to the World Health Organization criteria [1]. TNM (Tumor-Node-Metastasis) staging was done according to the 8th edition of the AJCC cancer staging manual [38]. The same data set was used in our previous studies [8,43,44], but clinical follow-up data were updated as of March 2020. All patients with total thyroidectomy took levothyroxine for suppression of thyroid-stimulating hormone (TSH). A total of 471 patients with PTC underwent radioactive iodine (RAI) remnant ablation after total thyroidectomy.

The therapeutic response was assessed by serum thyroglobulin (Tg), stimulated Tg, and anti-Tg antibody levels, imaging studies such as neck ultrasonography, and diagnostic RAI whole-body scan, or cytologic/histologic examination [6,45]. Excellent response to therapy was defined according to the 2015 ATA guidelines [6] and was based on a combination of the following characteristics: non-stimulated Tg level <0.2 ng/mL, stimulated Tg level <1 ng/mL, undetectable anti-Tg antibody, and negative imaging.

Patients who demonstrated an excellent response to therapy at the time of the last follow-up were considered NED at the final follow-up. A structural recurrence was defined as structural evidence of disease on imaging or biopsy-proven disease that was detected following any period of NED. Only biochemical evidence was not considered as recurrence in this study.

### 4.2. Tissue Microarray

Tissue microarrays were constructed from formalin-fixed, paraffin-embedded (FFPE) tissue blocks. The tissue microarray design was a 5 × 10 subarray with 2-mm cores at the 1-mm spacing. A 2-mm-diameter tissue core was punched out from a representative tumor area of each patient’s block and transplanted to a recipient block using a manual microarrayer (Quick-Ray set, Unitma, Seoul, Korea). Each tissue microarray block contained 47 cases of PTC, one case of normal thyroid tissue, one case normal tonsil tissue, and one case of placental tissue.

### 4.3. Immunohistochemistry

**Figure 5:**
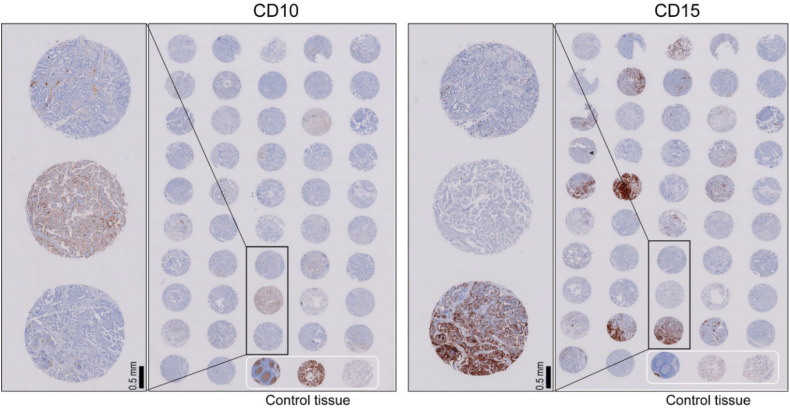
Immunohistochemical staining for CD10 and CD15 on tissue microarray. Tissue microarray consisted of 47 cores of tumor tissue and three cores of control tissue (normal tonsillar, placental, and thyroid tissues).

Serial 4-μm-thick sections were cut from tissue microarray blocks, deparaffinized, and rehydrated in serial graded ethanol washes. Immunohistochemistry for CD10 and CD15 was performed using an automated Dako OMNIS GI100 stainer (Dako, Agilent, Santa Clara, CA, USA) in accordance with the manufacturer’s instructions. Antigen retrieval was performed on sections for 30 min at 97 °C using the EnVision FLEX Target Retrieval Solution High pH (Dako). Tissue sections were incubated with mouse anti-human CD10 monoclonal antibody (1:50, clone 56C6, NCL-L-CD10-270, Novocastra, Newcastle Upon Tyne, UK) and mouse anti-human CD15 monoclonal antibody (1:1000, clone Carb-3, Dako) for 20 min, followed by visualization with Dako EnVision FLEX /HRP detection reagent for 20 min and substrate chromogen for 5 min. The specimens were then counterstained with hematoxylin for 3 min. For positive tissue controls, all tissue sections included tonsil and placental tissue (Figure 5). Normal thyroid tissue was used as negative tissue controls. One tissue section per each run was simultaneously incubated with the antibody diluent in place of the primary antibody.

### 4.4. Evaluation of Immunostaining

Two pathologists (Eun Ji Oh and Chan Kwon Jung) independently assessed CD10 and CD15 immunohistochemical staining. In the case of discrepancy, a consensus was reached between the two observers. Immunoreactivity of CD10 and CD15 was interpreted as the staining proportion of tumor cells. The cytoplasmic immunoreactivity for CD10 and CD15 in more than 5% of tumor cells was considered as positive [24].

### 4.5. BRAF Mutational Analysis

Genomic DNA was isolated from 10 μm-thick FFPE whole tissue sections enriched for the tumor cells by macrodissection using a RecoverAll Total Nucleic Acid Isolation Kit for FFPE (Life Technologies, ThermoFisher Scientific, Carlsbad, CA) according to the supplier’s instructions.

PCR reaction for the amplification of exon 15 of the *BRAF* gene was performed under the following conditions: 1 cycle of 3 min at 95 °C for denaturation, 35 cycles consisting of 30 s at 94 °C, 30 s at 55 °C, and 30 s at 72 °C, followed by a final cycle of 7 min at 72 °C. The PCR primers for BRAF were as follows: forward, 5’-TCATAATGCTTGC TCTGATAGGA-3’ and reverse, 5’-GGCCAAAAATTTAATCAGTGGA-3’. Sanger sequencing of PCR amplicons was performed using the same PCR primers as described previously [8,43].

### 4.6. TCGA Data Analysis

We obtained RNAseq-based, gene-level normalized RSEM (RNA-Seq by Expectation Maximization) scores for PTC (n = 505) from PanCancer Atlas resource (https://gdc.cancer.gov). The clinical, pathological, and molecular data for each TCGA sample were obtained from the article by the TCGA research network [46]. The expression levels of *MME* (*CD10*) mRNA and *FUT4* (*CD15*) mRNA were grouped into low (<median expression of the gene) and high expression (≥median expression) based on the median value of mRNA expression.

In the TCGA dataset, thyroid cancer staging was initially based on the 7th edition of the AJCC cancer staging system [46,47]. The extrathyroidal extension was classified into three categories, namely minimal (T3), moderately advanced (T4a), and very advanced (T4b). The minimal extrathyroidal extension refers to the extension to sternothyroid muscle or perithyroidal soft tissues. In the AJCC 8th edition, minor extension through the thyroid capsule seen only on histologic examination is not used for staging, and gross extrathyroidal extension involving strap muscles is classified into T3b disease. The presence of minor extrathyroidal extension involving strap muscle detected only by microscopy does not constitute T3b disease [38]. Although most T3 thyroid cancers by the AJCC 7th edition are reclassified into lower stages (T1 or T2) by the 8th AJCC staging system, some of them remain T3 with sternothyroid muscle invasion. We could not apply the 8th AJCC staging system in the TCGA dataset because there was no detailed information relating to cases with minimal extrathyroidal extension classified by the 7th AJCC system. Therefore, TNM staging in the TCGA dataset was done according to the 7th edition of the AJCC cancer staging manual [47].

### 4.7. Immunoprofiling of PTC in the TCGA Database

To infer the relative abundance of tumor infiltrating immune cells in PTC as we described previously [48], a support vector regression-based CIBERSORT algorithm was employed [30]. The default set (LM22) was used to estimate the relative abundance of 22 immune cell types. Ten cell types with positive and inverse correlation with *FUT4* expression were selected for demonstration. A cytolytic score representing the activity of immune cytolytic effectors was calculated as the geometric means of expression of *GZMA* and *PRF1* as previously described [36]. We used ESTIMATE R packages to estimate the score representing the proportion of immune and stromal cells in PTC [29]. Single sample gene set enrichment analysis was performed to estimate the expression-based activity of 50 hallmark gene sets as available in MSigDB Collections (https://www.gsea-msigdb.org/gsea/msigdb/collections.jsp).

### 4.8. Statistical Analysis

Chi-square test and Fisher’s exact test were used to analyze the association between CD10 and CD15 protein expression and clinicopathologic features. Survival curves were plotted using the Kaplan–Meier method. Statistical differences between survival curves were calculated using the log-rank test. Cox proportional-hazard model was employed for multivariate analysis of recurrence-free survival rates. Univariate and multivariate logistic regression analyses of variables were performed to determine whether clinicopathological variables were significantly associated with long-term clinical outcomes. The independent variables were entered simultaneously using the enter method.

All statistical analyses were performed by IBM SPSS Statistics for Windows, Version 21.0. (IBM Corp., Armonk, NY, USA) and GraphPad Prism (version 6.05, GraphPad Software, La Jolla, CA, USA). A two-sided *p* value < 0.05 was considered as statistically significant.

## 5. Conclusions

The expression of CD10 and CD15 might be implicated in the early stage of PTC development. CD15 expression was an independent prognostic marker for improved recurrence-free survival and excellent clinical outcomes after treatment, but CD10 expression had no impact on prognosis in PTC patients. The good therapeutic response to RAI therapy in PTC patients with CD15 expression might be attributed to the increased NIS expression.

## Figures and Tables

**Table 1:** Relationship between clinicopathologic characteristics and the expression of CD10 and CD15 in 515 patients with papillary thyroid carcinoma.

	n	CD10 Expression			CD15 Expression		
		Positive	Negative	*p* Value	Positive	Negative	*p* Value
Age (years)				0.772			0.233
<55	365	141 (38.6%)	224 (61.4%)		203 (55.6%)	162 (44.4%)	
≥55	150	60 (40.0%)	90 (60.0%)		92 (61.3%)	58 (38.7%)	
Sex				0.049			0.009
Female	405	167 (41.2%)	238 (58.8%)		244 (60.2%)	161 (39.8%)	
Male	110	34 (30.9%)	76 (69.1%)		51 (46.4%)	59 (53.6%)	
Histologic type				0.009			0.002
Classic	453	174 (38.4%)	279 (61.6%)		260 (57.4%)	193 (42.6%)	
Follicular variant	17	2 (11.8%)	15 (88.2%)		3 (17.6%)	14 (82.4%)	
Tall cell variant	21	10 (47.6%)	11 (52.4%)		15 (71.4%)	6 (28.6%)	
Other	24	15 (62.5%)	9 (37.5%)		17 (70.8%)	7 (29.2%)	
Extrathyroidal extension							
Absent	137	38 (27.7%)	99 (72.3%)		64 (46.7%)	73 (53.3%)	
Minimal *	279	120 (43.0%)	159 (57.0%)	0.003	166 (59.5%)	113 (40.5%)	0.014
Gross	99	43 (43.4%)	56 (56.6%)	0.317	65 (65.7%)	34 (34.3%)	0.061
Pathological (p) T stage				0.164			0.181
pT1	357	129 (36.1%)	228 (63.9%)		197 (55.2%)	160 (44.8%)	
pT2	56	27 (48.2%)	29 (51.8%)		30 (53.6%)	26 (46.4%)	
pT3	81	34 (42.0%)	47 (58.0%)		55 (67.9%)	26 (32.1%)	
pT4	20	10 (50.0%)	10 (50.0%)		12 (60.0%)	8 (40.0%)	
Lymph node metastasis				0.143			0.735
Absent	197	69 (35.0%)	128 (65.0%)		111 (56.3%)	86 (43.7%)	
Present	318	132 (41.5%)	186 (58.5%)		184 (57.9%)	134 (42.1%)	
Distant metastasis				0.286			0.062
Absent	504	195 (38.7%)	309 (61.3%)		292 (57.9%)	212 (42.1%)	
Present	11	6 (54.5%)	5 (45.5%)		3 (27.3%)	8 (72.7%)	
AJCC stage 8th				0.229			0.646
Stage I	421	160 (38.0%)	261 (62.0%)		240 (57.0%)	181 (43.0%)	
Stage II	81	34 (42.0%)	47 (58.0%)		49 (60.5%)	32 (39.5%)	
Stage III	9	5 (55.6%)	4 (44.4%)		6 (66.7%)	3 (33.3%)	
Stage IV	4	2 (50.0%)	2 (50.0%)		0	4 (100.0%)	
Recurrence				0.656			0.034
Absent	461	179 (38.8%)	282 (61.2%)		270 (58.6%)	191 (41.4%)	
Present	45	19 (42.2%)	26 (57.8%)		19 (42.2%)	26 (57.8%)	
*BRAF* ^V600E^				0.011			<0.001
Absent	83	22 (26.5%)	61 (73.5%)		13 (15.7%)	70 (84.3%)	
Present	432	179 (41.4%)	253 (58.6%)		282 (65.3%)	150 (34.7%)	

AJCC, American Joint Committee on Cancer. The AJCC 8th edition cancer staging was used in this study. * Minimal extrathyroidal extension by AJCC 8th edition refers to the extension to perithyroid adipose tissue, strap muscles, nerves, or small vascular structures identified only by microscopic examination [27].

**Table 2:** Multivariate Cox regression analysis of recurrence-free survival in 467 patients with papillary thyroid carcinoma treated with total thyroidectomy and radioiodine remnant ablation.

Variable	Adjusted Hazard Ratio	95% CI	*p* Value
Age (years), ≥55 vs. <55	0.900	0.443–1.830	0.900
Sex, male vs. female	0.936	0.475–1.844	0.848
Histologic subtype, aggressive vs. non-aggressive	1.395	0.424–4.594	0.584
Pathological T stage, pT3-4 vs. pT1-2	1.825	0.972–3.426	0.061
Lymph node metastasis, present vs. absent	7.026	2.167–22.786	0.001
CD15 expression, present vs. absent	0.500	0.274–0.911	0.024

Aggressive histologic subtype includes 21 tall cell variants, two hobnail variants, and one solid variant. CI: confidence interval.

**Table 3:** Univariate and multivariate logistic regression analyses of clinicopathological variables associated with long-term excellent therapeutic response in 467 patients with papillary thyroid carcinoma treated with total thyroidectomy and radioiodine remnant ablation.

Variable	Univariate Analysis		Multivariate Analysis	
	OR (95% CI)	*p* Value	Adjusted OR (95% CI)	*p* Value
Age (years), ≥55 vs. <55	0.991 (0.427–2.297)	0.982	0.985 (0.415–2.339)	0.973
Sex, male vs. female	0.708 (0.304–1.649)	0.423	0.822 (0.345–1.957)	0.657
Histologic subtype, aggressive vs. non-aggressive	0.478 (0.128–2.624)	0.478	0.683(0.139–3.350)	0.638
Pathologic T stage, pT3-4 vs. pT1-2	0.336 (0.155–0.731)	0.006	0.298 (0.135–0.658)	0.003
CD15 expression, present vs. absent	2.368 (1.092–5.134)	0.029	2.678 (1.215–5.902)	0.015

All patients without lymph node metastasis achieved an excellent therapeutic response. Aggressive histologic subtype includes 21 tall cell variants, two hobnail variants, and one solid variant. OR, odds ratio; CI: confidence interval.

**Table 4:** Relationship between clinicopathologic characteristics and the expression of *MME (CD10)* and *FUT4 (CD15)* mRNA expression in 454 patients with papillary thyroid carcinoma in The Cancer Genome Atlas (TCGA) database.

Variable	*n*	*MME* (*CD10*) mRNA			*FUT4* (*CD15*) mRNA		
		High	Low	*p* Value	High	Low	*p* Value
Age (years)	454			0.657			0.001
<45	211	106 (50.2%)	105 (49.8%)		120 (56.9%)	91 (43.1%)	
≥45	243	117 (48.1%)	126 (51.9%)		101 (41.6%)	142 (58.4%)	
Sex	454			0.765			0.279
Female	123	59 (48.0%)	64 (52.0%)		65 (52.8%)	58 (47.2%)	
Male	331	164 (49.5%)	167 (50.5%)		156 (47.1%)	175 (52.9%)	
Histologic type	454			0.410			0.031
Classic	312	172 (55.1%)	140 (44.9%)		165 (52.9%)	147 (47.1%)	
Follicular	99	17 (17.2%)	82 (82.8%)		36 (36.4%)	63 (63.6%)	
Tall cell	34	30 (88.2%)	4 (11.8%)		18 (52.9%)	16 (47.1%)	
Other	9	4 (44.4%)	5 (55.6%)		2 (22.2%)	7 (77.8%)	
Extrathyroidal extension	441						
Absent	309	130 (42.1%)	179 (57.9%)		146 (47.2%)	163 (52.8%)	
Minimal (T3) *	117	77 (65.8%)	40 (34.2%)	<0.001	63 (53.8%)	54 (46.2%)	0.224
Advanced (T4)	15	11 (73.3%)	4 (26.7%)	0.060	5 (33.3%)	10 (66.7%)	0.231
Pathologic (p) T stage	452			0.001			0.356
pT1-T2	283	122 (43.1%)	161 (56.9%)		133 (47.0%)	150 (53.0%)	
pT3-T4	169	100 (59.2%)	69 (40.8%)		87 (51.5%)	82 (48.5%)	
pN stage	409			<0.001			0.154
pN0	208	82 (39.4%)	126 (60.6%)		94 (45.2%)	114 (54.8%)	
pN1	201	124 (61.7%)	77 (38.3%)		105 (52.5%)	96 (47.8%)	
M stage	250			1.000			0.148
M0	242	128 (52.9%)	114 (47.1%)		133 (55.0%)	109 (45.0%)	
M1	8	4 (50.0%)	4 (50.0%)		2 (25.0%)	6 (75.0%)	
AJCC stage 7th	452			0.007			0.428
Stage I–II	309	139 (45.0%)	170 (55.0%)		155 (50.2%)	154 (49.8%)	
Stage III–IV	143	84 (58.7%)	59 (41.3%)		66 (46.2%)	77 (53.8%)	
Recurrence risk group	442			<0.001			0.023
Low	166	56 (33.7%)	110 (66.3%)		78 (47.0%)	88 (53.0%)	
Intermediate	252	148 (58.7%)	104 (41.3%)		134 (53.2%)	118 (46.8%)	
High	24	14 (58.3%)	10 (41.7%)		6 (25.0%)	18 (75.0%)	
*BRAF*-*RAS* signature	391			<0.001			0.002
*BRAF*-like	272	177 (65.1%)	95 (34.9%)		151 (55.5%)	121 (44.5%)	
*RAS*-like	119	16 (13.4%)	103 (86.6%)		46 (38.7%)	73 (61.3%)	

AJCC, American Joint Committee on Cancer. The AJCC 7th edition cancer staging was used in the TCGA dataset. * Minimal extrathyroidal extension by AJCC 7th edition refers to the extension to sternothyroid muscle or perithyroid soft tissues [28].
